# Model Selection in Historical Research Using Approximate Bayesian Computation

**DOI:** 10.1371/journal.pone.0146491

**Published:** 2016-01-05

**Authors:** Xavier Rubio-Campillo

**Affiliations:** Computer Applications in Science & Engineering department, Barcelona Supercomputing Centre, Barcelona, Spain; New York State Museum, UNITED STATES

## Abstract

**Formal Models and History:**

Computational models are increasingly being used to study historical dynamics. This new trend, which could be named Model-Based History, makes use of recently published datasets and innovative quantitative methods to improve our understanding of past societies based on their written sources. The extensive use of formal models allows historians to re-evaluate hypotheses formulated decades ago and still subject to debate due to the lack of an adequate quantitative framework. The initiative has the potential to transform the discipline if it solves the challenges posed by the study of historical dynamics. These difficulties are based on the complexities of modelling social interaction, and the methodological issues raised by the evaluation of formal models against data with low sample size, high variance and strong fragmentation.

**Case Study:**

This work examines an alternate approach to this evaluation based on a Bayesian-inspired model selection method. The validity of the classical Lanchester’s laws of combat is examined against a dataset comprising over a thousand battles spanning 300 years. Four variations of the basic equations are discussed, including the three most common formulations (linear, squared, and logarithmic) and a new variant introducing fatigue. Approximate Bayesian Computation is then used to infer both parameter values and model selection via Bayes Factors.

**Impact:**

Results indicate decisive evidence favouring the new fatigue model. The interpretation of both parameter estimations and model selection provides new insights into the factors guiding the evolution of warfare. At a methodological level, the case study shows how model selection methods can be used to guide historical research through the comparison between existing hypotheses and empirical evidence.

## Introduction

The discipline of History presents its ideas as descriptive models expressed in natural language. Historians use the flexibility of this communication system to explain the complexity and diversity of human societies though their written records. The approach is different than the majority of scientific disciplines, which formulate their theories in formal languages such as mathematics. Formal languages are not as flexible as natural languages, but they are much better defining concepts and relations without ambiguities [1]. Hypotheses defined in formal language can then be falsified against empirical evidence, and quantitative methods can then be applied to compare predictions generated by a theory to observed patterns. As a consequence, an old theory can be replace by a new one when it has superior explanatory power.

This evaluation of ideas does not happen in History. Quantitative methods cannot be used to falsify descriptive models or perform cross-temporal and cross-spatial comparison. These inabilities are central to the current methodological debates of the discipline [2–6]. There is a clear desire to identify both the common trajectories and the observed differences between case studies with diverse spatiotemporal coordinates. However, it is unclear how this could be achieved using the common methods of the discipline.

A possible approach to tackle this challenge is to shift the discipline from descriptive to formal models [7]. This innovation could allow historians to know under what extent a working hypothesis explains a historical dynamic by quantifying the distance between the predictions of a model and the patterns observed in the evidence. This new approach has clear benefits, but it is not an easy task as it requires a) formal models, b) quantified datasets and c) methods to compare both components.

These debates are intrinsically linked with the increasing number of available databases and historical research using formal models [8–10]. The rise of what we could define as *Model-Based History* is changing the way researchers study historical trajectories [2]. To date, this new approach to the past has been focused on three main topics: trade networks [11], sociocultural evolution [7] and warfare [12–14]). The increase in the number of works is diversifying the topics examined by *Model-Based History*, and now it includes fields such as knowledge exchange [15] or the evolution of religion [16].

Quantitative comparison between models and observations is one of the advantages of this new approach. The most common statistical framework to perform this evaluation is Null Hypothesis Significance Testing. First, the problem to solve is defined as a clear research question and a working hypothesis *H*1. This hypothesis is a possible answer which could be falsified by existing evidence. The explanation provided by *H*1 will then compete against a null hypothesis *H*0. *H*0 is an alternative that does not take into account *H*1. *H*1 is translated into a formal model, usually a computer simulation of the dynamics encapsulated in the hypothesis. *Model-Based History* often prefers bottom-up techniques such as Agent-Based Models [17] or complex network analysis [18]. Classical equation-based models are also applied, but these innovative approaches seem better suited to the type of social processes examined by the discipline. The created model defines the system at a small-scale level (e.g. individual or groups), and it evolves through the interaction between these entities. The emergence of distinctive large-scale patterns generated by this set of interactions is then compared to empirical data. If the probability of getting the observed patterns without *H*1 is less than a given confidence interval (i.e. the *p-value*) we can reject *H*0, thus accepting *H*1.

Null Hypothesis Significance Testing is useful to prove that our model has higher predictive power than a random process. However, it is not designed to compare multiple potentially valid explanations. Model selection is a different approach designed to quantify by how much a model is better at explaining evidence than alternate models. Model selection is having increasing popularity due to the current debates on the use of statistics analysis for scientific research [19–23]. It is worth mentioning that neither method seems better than the other one, and the choice will depend on the aim of the research: Null Hypothesis Significance Testing aims to know if the observed process could be explained without the working hypothesis, while model selection aims to choose which hypothesis is better at matching evidence.

The model selection approach provides a set of new methods to evaluate models. Most of them quantify the loss of information from each model to the evidence using information criteria [24]. Two of the most widely used methods are Akaike Information Criterion and Bayesian Information Criterion. Both of them fit the different models to the observed patterns using maximum likelihood methods, and then they calculate an index of information loss (i.e. low values indicate better models).

A different solution is to use the Bayesian statistical framework. It is based on the idea that the knowledge of a given system with uncertainty can be gradually updated through new evidence. The process is achieved by computing the probability that a given hypothesis is correct, considering both existing knowledge and new data. The main advantage of this approach is that it seems better fitted to evaluate competing models under high levels of uncertainty and equifinality [25]. Despite its interest, scientific research did not start using the Bayesian framework until recent years, even if it was formulated 200 years ago [26]. The delay on the adoption was mainly caused by the mathematical complexities of applying Bayesian statistics to non-trivial problems. The development of new computational methods such as Markov Chain Monte-Carlo and Approximate Bayesian Computation (ABC) has mainly solved this limitation, thus explaining the current success of Bayesian inference.

Historians constantly deal with competing explanations of uncertain datasets, so it seems that Bayesian model selection can be useful to the discipline. This potential can also be inferred from the fact that other historical disciplines such as biology and archaeology are part of this Bayesian renaissance. Biology is particularly active in using these methods in fields such as population genetics and ecology [27, 28]. Archaeology traditionally limited Bayesian inference to C14 dates [29], but model selection techniques are becoming popular beyond this application [30–33]. These examples suggest that Bayesian model selection can be applied to History, considering the similarities between the three disciplines. First, all these fields study temporal trajectories using data with high levels of uncertainty. Second, the analysis of these datasets implies that they need to evaluate the plausibility of multiple competing hypotheses. Finally, all of them want to identify patterns generated as an aggregate of individual behaviour. As a consequence, it seems clear that Bayesian model selection would have significant utility for historians.

This paper presents the use of Bayesian inference to perform model selection in historical research. The utility of a Bayesian-inspired computational method known as Approximate Bayesian Computation is discussed. The use of ABC is then illustrated with a classical example of formal model used in History: the classical Lanchester’s laws of warfare. Next section presents the case study, the model selection framework and the competing models. Third section shows the results of the method, both in terms of model selection and parameter estimation. The text then interprets these results and concludes with an evaluation of the approach in the context of *Model-Based History*.

## Materials and Methods

### Case study: the evolution of combat

Warfare is probably the first human activity ever explored with formal models. Their use began in early XIXth century in the form of boardgames such as *kriegsspiel*. They were used to train officers on managing armies and fighting the enemy. These practices had a major impulse during Second World War with the creation of *Operations Research*. This new research field focused on developing formal models able to help commanders on decision-making [34]. The introduction of the first computers expedited the use of these quantitative methods during the Cold War, establishing them as a standard procedure for training and planning. In contrast, History is only now incorporating some of these techniques to the study of past conflicts [35]. Boardgames, mathematical models and computer simulations are proving their utility in the task of studying warfare understood as an unfortunate part of human culture [36].

The theoretical model formulated by F.W. Lanchester in 1916 is one of the most popular mathematical formulations used in the field [37]. Lanchester aimed to design the laws predicting the casualties of two enemy forces engaged in land battle. He proposed a system of coupled differential equations where casualties were dependent on two factors: a) force size and b) fighting value. The first factor takes into account the importance of sheer numbers on the outcome of military conflict, while the second factor encapsulates qualitative differences between individual fighting skills (e.g. morale, training, technology, etc.). Two models were initially proposed: the *linear law* and the *square law*. The *linear law* aimed to capture the dynamics of ancient battles, where the supremacy of hand-to-hand combat meant that each soldier could only attack an opponent at a given moment. The equations defining the rate of casualties in a battle between armies Blue and Red are defined in Eq 1:

$$\begin{array}{c} \frac{dB}{dt}=-rBR\;\frac{dR}{dt}=-bRB \end{array}$$ (1)

with *B, R* as the size of the forces and *r, b* as their fighting value. The rate of casualties is proportional to both sizes, so even highly disproportionate odds would cause similar casualties to both opponents.

The *square law* models warfare after the introduction of gunpowder-based weapons. This technological innovation increased the range, thus allowing each soldier to attack multiple enemies. The *squared law* models the casualties of a force as the enemy’s force size multiplied by the fighting value of its individuals, as seen in Eq 2:

$$\begin{array}{c} \frac{dB}{dt}=-rR\;\frac{dR}{dt}=-bB \end{array}$$ (2)

The Lanchester’s laws generated a large amount of interest during the Cold War [38–42]. The debate was centred on the actual predictive power of the laws, and it included the formulation of alternate proposals such as the popular *logarithmic* model. It suggested that the casualties suffered by a force are not dependant on the enemy’s size, but on its own size as defined in Eq 3 [40]:

$$\begin{array}{c} \frac{dB}{dt}=-rB\;\frac{dB}{dt}=-bR \end{array}$$ (3)

Several works discussed the validity of the laws [42, 43]. Other contributions extended the original framework introducing concepts such as spatial structure or system dynamics [44, 45]. The utility of the model was also expanded beyond its initial purpose, and has been successfully applied to study competition dynamics in ecology [46–48], evolutionary biology [49] or economics [50].

Model selection were also applied to compare the plausibility of these models against historical evidence. The most extensive effort was made by Charles D. Allen’s in his monograph [51]. The author tested the validity of different models to explain a dataset of 1080 land battles from the middle of XVIIth century to the beginnings of the XXth century. The analysis suggested that the logarithmic model has higher explanatory power than the two classical models. However, the coarse-grained results assumed that this power remained constant during the whole period, thus not examining the validity of the models for the different phases of warfare. Similar works used Bayesian inference to evaluate the Lanchester’s laws in specific scenarios. They included biological case studies [48], daily casualties during Inchon-Seoul campaign in 1950 [52] or attrition during the battle of the Ardennes in 1944 [53, 54].

All these results suggests that the Lanchester’s laws are useful to understand if casualties are more influenced by quantitative or qualitative factors. Some authors suggested that the models should introduce dynamic parameters such as variable fighting values or fatigue [44]. However, as some of these works highlights, a pure Bayesian framework could hardly cope with the mathematical difficulties added by this new complexity.

#### The dataset

The dataset used in this study is based on Allen’s list of battles, originally compiled in a previous work [55]. The introduction of weapons with longer ranges over 300 years should be reflected in a gradual increase in the validity of the *squared* model over the *linear* model. In order to test this idea the span has been divided in four periods, based on prior opinions of decisive transitions in the evolution of warfare [56]:

1. *Pike and Musket (1620–1701)*. The first period was characterised by deep formations of soldiers (i.e. *tercios* and regiments) armed with muskets and pikes.
2. *Linear warfare (1702–1792)*. The War of the Spanish Succession (1702–1714) saw a shift in battle tactics and technological innovations. Armies were deployed in thin formations exclusively armed with muskets, while pikes were substituted by bayonets.
3. *Napoleonic Wars (1793–1860)*. The French Revolution forced another major transition in warfare, which was mainly adopted during the Napoleonic wars. The new concept of *citizen* armies allowed the states to increase the size of their forces up to the limits imposed by pre-industrial logistics.
4. *American Civil War (1861–1905)*. The impact of industry development became explicit on the battlefield during the American Civil War. The size of armies and the lethality of their weapons steadily increased until fully industrialised armies were deployed in the Russo-Japanese War. This conflict was the prelude of what would be seen during the two world wars.

**Fig 1:**
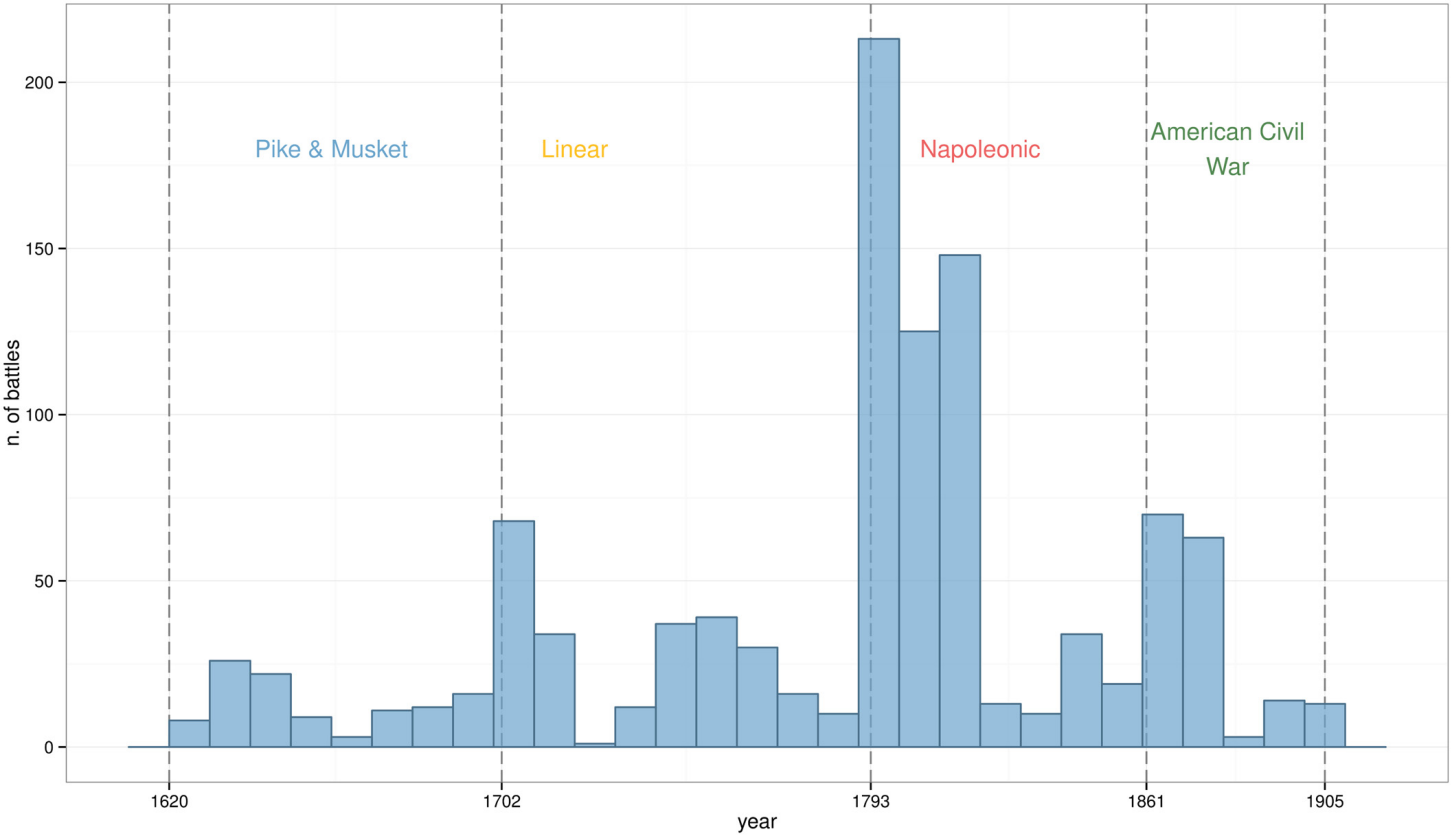
Number of battles by decade. The three identified transitions correlate with periods of intensive warfare.

**Fig 2:**
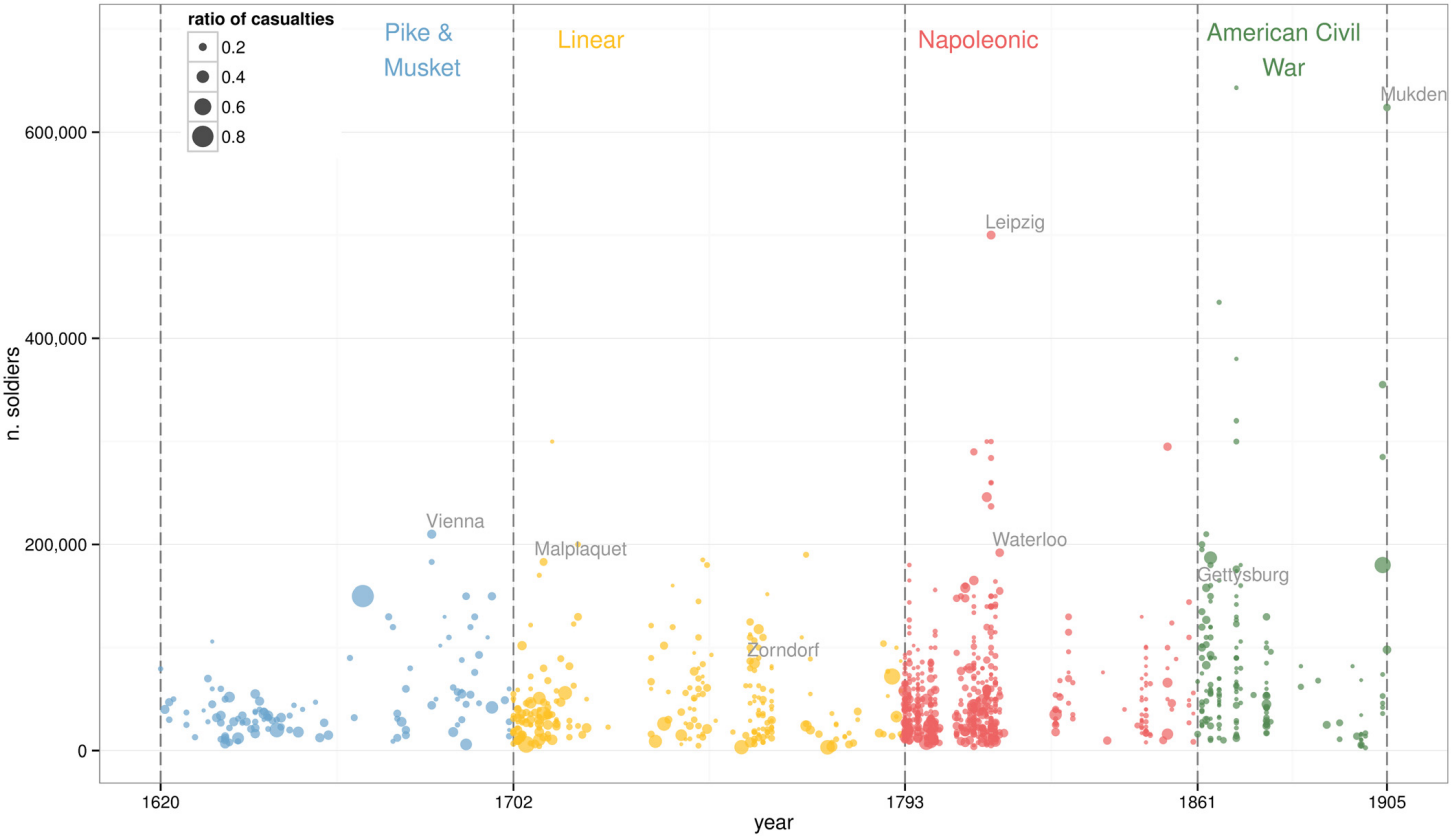
Size and casualty ratio by battle. The total number of soldiers involved in each battle is defined in the Y axis while the size of each point shows the casualty ratio of the battle.

Exploratory Data Analysis has been used to identify structural patterns in the dataset. A time series of the number of battles can be seen in Fig 1, while size and casualty ratios are depicted in Fig 2. These visualisations shows how the dataset has relative small sample size and high variance. These are common properties seen in historical data. The figures suggest that the number of battles remained constant during the 300 years with the exception of the Napoleonic Wars. At the same time, the gradual increase on average army size seems linked to a decrease on casualty ratios.

### The model selection framework

Standard Bayesian inference updates a set of prior beliefs considering new evidence and a given likelihood function. Prior beliefs aggregate the existing knowledge of a given topic, and the degree of credibility of this knowledge. These beliefs are translated into parameters of the model. The possible values for each parameter receive an initial probability following a specific statistical distribution. The likelihood function is used to compute the probabilities of any given result considering the value of the input parameters. The updated knowledge (i.e. the *the posterior distribution*) is then computed following Bayes’ rule:

$$\begin{array}{c} P\left(\theta |D\right)=\frac{P(D|\theta )\cdot P(\theta )}{P(D)} \end{array}$$

being *θ* the considered value and *D* the observed data. This can be translated as (following [57]):

$$\begin{array}{c} posterior=\frac{likelihood\cdot prior}{evidence} \end{array}$$

A barrier to the adoption of Bayesian inference is the difficulty to derive likelihood functions when the examined model is not a standard statistical distribution. This constraint limits the use of the framework for computer simulations encapsulating complex dynamics such as the ones explored in *Model-Based History*. A major breakthrough to this issue is the recent development of ABC [58, 59].

ABC comprises a family of computationally-intensive algorithms able to approximate posterior distributions without using likelihood functions. These methods identify the regions of the prior space producing the closest results to the evidence. This capability of extending the Bayesian framework to any computer simulation has exponentially increased the popularity of ABC during the last decade, including the other historical disciplines: biology [60–63], and archaeology [31, 33, 64, 65]).

The analysis performed in this work implements the simplest ABC method: the rejection algorithm [66]. It is not the most efficient ABC method (see [67, 68] for alternatives), but its simplicity and lack of assumptions makes it perfect for illustrative purposes. It is defined as follows:

1. Initialise parameters sampling the prior distributions
2. Run the model and compute the distance to evidence
3. If distance is within the closest runs below a tolerance level *τ* keep values of parameters; otherwise discard them.

This algorithm is executed a large number of runs, and the set of kept parameter values is used as the posterior distribution.

### Definition of competing models

We will evaluate the plausibility of four different variations of the Lanchester equations: the two original laws (*linear* and *squared*), the popular *logarithmic* variation and a new model adding *fatigue* effects. For convenience the models have been here transformed to difference equations as seen in Eqs 4, 5 and 6:

Linear:

$$\begin{array}{c} \begin{array}{c} B_{t+1}=B_{t}-rB_{t}R_{t}\\ R_{t+1}=R_{t}-bR_{t}B_{t} \end{array} \end{array}$$ (4)

Squared:

$$\begin{array}{c} \begin{array}{c} B_{t+1}=B_{t}-rR_{t}\\ R_{t+1}=R_{t}-bB_{t} \end{array} \end{array}$$ (5)

Logarithmic:

$$\begin{array}{c} \begin{array}{c} B_{t+1}=B_{t}-rB_{t}\\ R_{t+1}=R_{t}-bR_{t} \end{array} \end{array}$$ (6)

The fourth model adds fatigue to the logarithmic model. This factor is modelled as a gradual decrease in the efficiency of the armies as defined in Eq 7):

Fatigue:

$$\begin{array}{c} \begin{array}{c} B_{t+1}=B_{t}-\frac{rB_{t}}{log(e+t)}\\ R_{t+1}=R_{t}-\frac{bR_{t}}{log(e+t)} \end{array} \end{array}$$ (7)

Fighting value *b* is scaled to the maximum number of casualties that *B* can inflict to *R* in a time step. In order to avoid disparate values *b* is defined following Eq 8 for the *linear* model and Eq 9 for the other three.

Linear law:

$$\begin{array}{c} b=\frac{100}{B_{t=0}\cdot R_{t=0}} \end{array}$$ (8)

Other models:

$$\begin{array}{c} b=\frac{100}{max(B_{t=0},R_{t=0})} \end{array}$$ (9)

The enemy’s fighting value *r* is then defined as *b* multiplied by an odds ratio *P*. In this way the individual value of a Red soldier is expressed as a ratio of Blue’s value (e.g. *P* = 2 would mean that each Red soldier is as lethal as two Blue soldiers).

**Fig 3:**
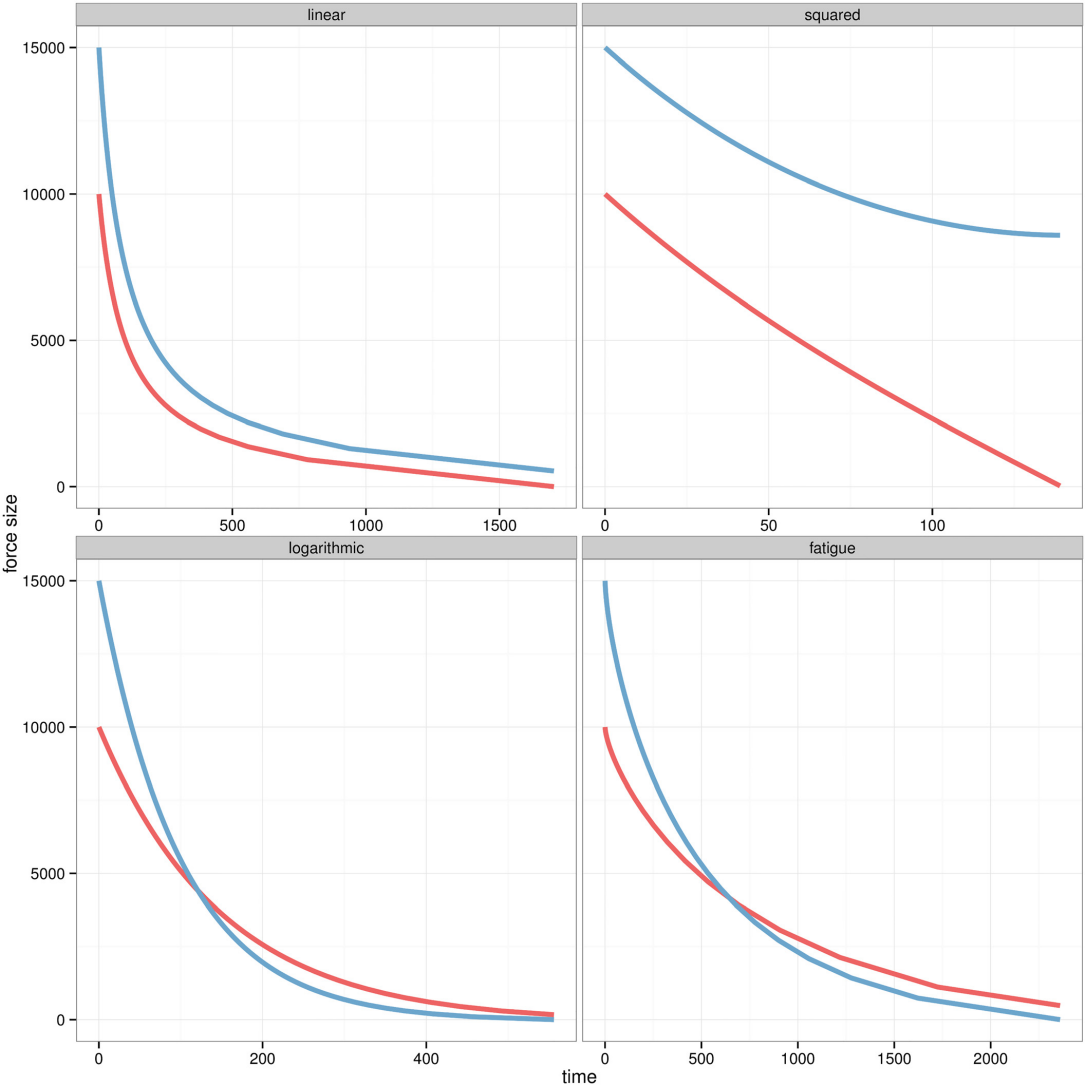
Time series of attrition. Casualties of two forces as computed by the four competing models with *P* = 1.5, *B*_*t* = 0_ = 15000 and R_*t* = 0_ = 10000.

Distinctive dynamics for each model are observed in Fig 3. All models are initialised as a battle where an army is being opposed by a smaller force with higher fighting value. In the *linear* model the forces have similar casualty rates, while size has a bigger impact in the *squared* model. The *logarithmic* model increases the weight of fighting value over size as the smaller force finishes with more soldiers. The *fatigue* model generates similar casualties than the *logarithmic* model, but they are distributed over a longer period of time.

### Experiment Design

**Fig 4:**
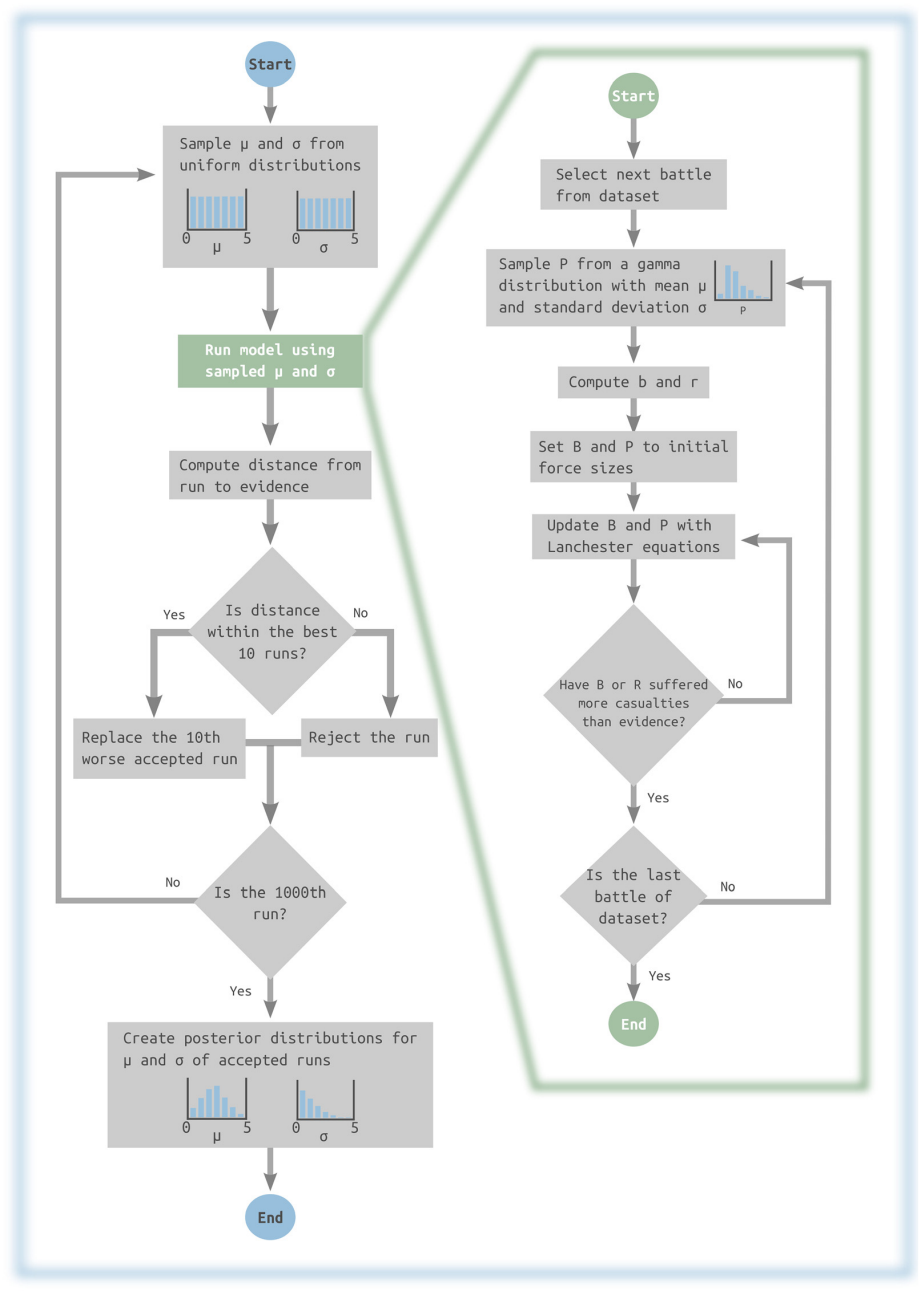
Flowchart for the ABC framework. Example for a experiment using 1000 runs and tolerance τ = 0.01. Left side illustrates the rejection algorithm while the green panel details the simulation of the Lanchester model.

Previous authors suggested that the deterministic nature of the original laws was too rigid to perform a proper comparison with long-term observations. Using a fixed *P* for a large number of battles would ignore any slight variation on the fighting value odds from one engagement to the next one. The issue has been solved introducing stochasticity in *P*, which is sampled every battle from a gamma distribution with shape *κ* and scale *θ*. For convenience the input parameters are expressed as mean *μ* and standard deviation *σ*, which are then used to compute $\kappa =\;{\left(\frac{\mu }{\sigma }\right)}^{2}$ and $\theta =\frac{\sigma^{2}}{\mu }$. The outcome of each battle is generated using as parameters the sampled *P* and initial army sizes *B*_*t* = 0_, *R*_*t* = 0_ set to historical values. The chosen Lanchester variant as defined in Eqs 4–7 is then iterated until one of the forces has suffered as many casualties as recorded in the historical data. The entire workflow is depicted in Fig 4.

The rejection algorithm calculates a distance between the results of a single run and observations. A popular approach is the comparison of summary statistics aggregating the outcome of a run against the evidence. However, this solution has theoretical issues which are currently being discussed [69]. This experiment avoids the debate by directly comparing the set of casualties for each battle and side. The distance between a simulation run and evidence is the absolute difference between simulated and historical casualties divided by historical casualties, thus normalising the weight of all battles regardless their total size. This comparison is performed identifying both in the evidence and simulation the Red army *R* as the side with lower casualty ratio in each battle.

Uninformed prior beliefs were used for the two parameters (*μ* and *σ*). The limits of their uniform distributions were defined as U(0,5), based on Allen’s results. Each competing model was ran 1 million times for each period. Sensitivity to tolerance levels was accounted by storing posterior distributions for different thresholds (*τ* = 0.05, *τ* = 0.005 and *τ* = 0.0005).

The model selection method is based on Bayes Factors. They quantify the relative likelihood of different competing models against the evidence expressed as an odds ratio [70]. This ratio was quantified with the common method of introducing a third parameter *m* as a model index variable [59]. It was used within a hierarchical model where *m* identified which of the four variants of the Lanchester’s laws was used during the run. Bayes Factors are then computed as the posterior distribution of *m* within the tolerance level *τ*.

## Results

### Model selection

**Fig 5:**
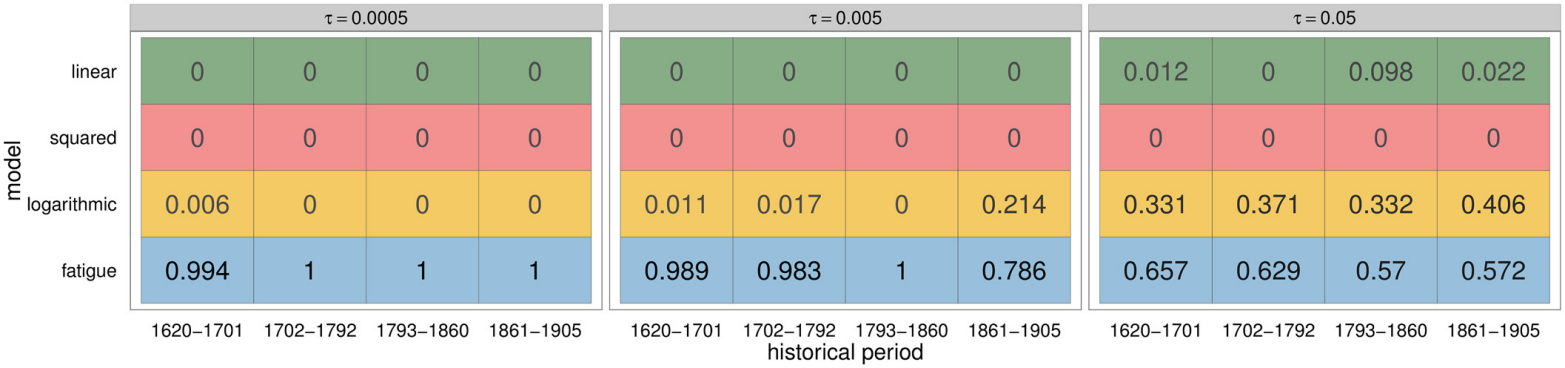
Model selection for different tolerance levels. Proportion of the models used in the best runs for the four historical periods and three *τ* values (corresponding to the selection of left: 500, centre: 5000 and right: 50000 best runs).

The *fatigue* model is decisively selected for all periods when using the lowest *τ* = 0.0005 (see Fig 5 left). The two original models (*linear* and *squared*) are not present in this set comprising the best 500 runs, while the logarithmic is only present for the XVIIth century. Larger tolerance levels increase the relevance of the *linear* and *logarithmic* models, while the squared model is never selected.

**Fig 6:**
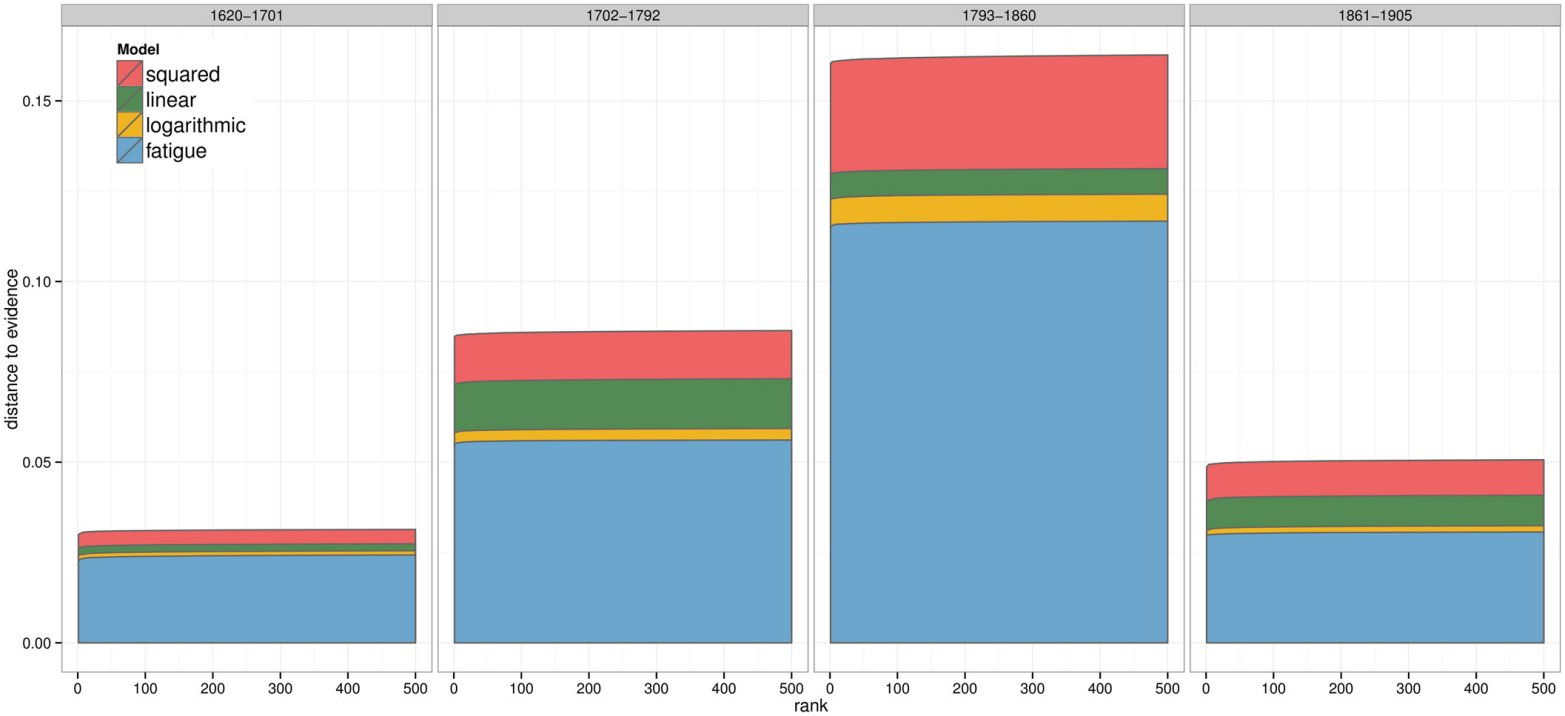
Distance from fatigue model to evidence with *τ* = 0.0005. Absolute distances (Y axis) of the best 500 runs ordered by rank (X axis, being 1 the best one), model (colour) and historical period (left to right).

The estimation of distances in Fig 6 shows that the plausibility of the models is not constant over the different periods. The four models followed the same trend, as their ranks remain constant over the different phases. In addition, all of them performed much worse for the battles of the third period (i.e. Napoleonic wars).

### Parameter estimation

**Fig 7:**
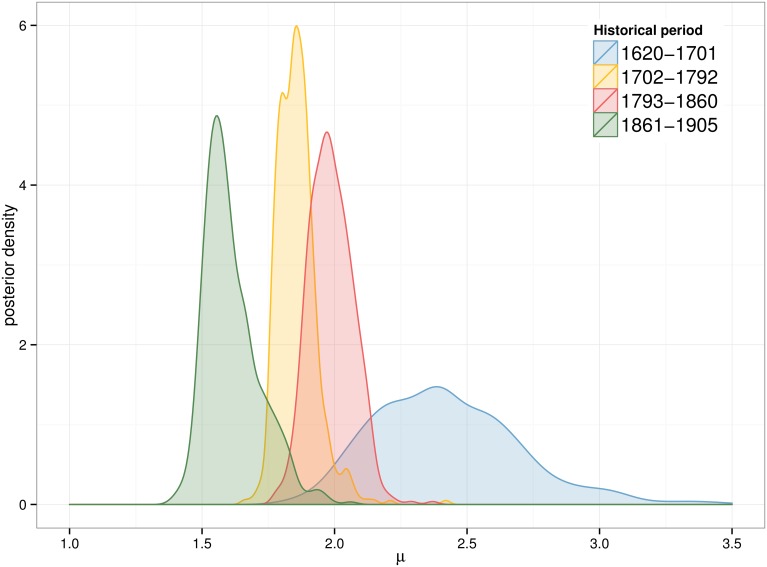
Posterior distribution of *μ*. Results for the *fatigue* model and *τ* = 0.0005.

**Fig 8:**
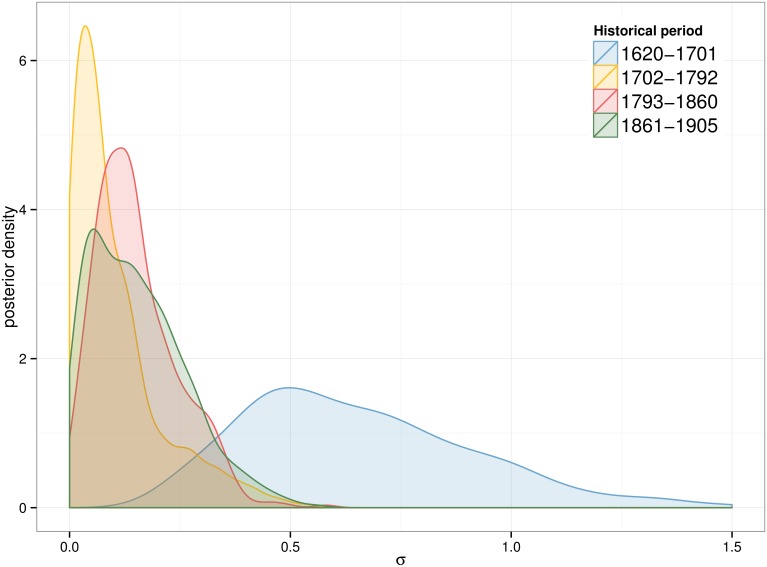
Posterior distribution of *σ*. Results for the *fatigue* model and *τ* = 0.0005.

The posterior distribution for parameters *μ* and *σ* is now examined for the *fatigue* model at *τ* = 0.0005. Fig 7 and Fig 8 show that both parameters follow unimodal distributions for all periods. The complete set of posterior distributions can be observed in SI 1, where similar patterns are observed for the other models (see S1 Fig for parameter *μ* and S2 Fig for parameter *σ*).

**Fig 9:**
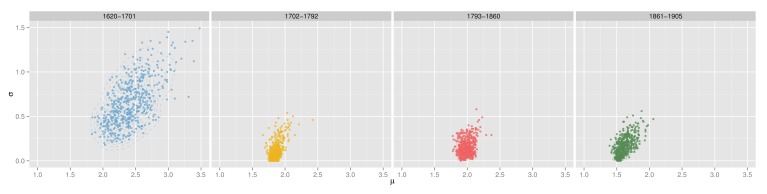
Relation between *μ* and *σ* values with *τ* = 0.0005. Large *σ* values are only selected when *μ* value is also large.

The parameter *μ* exhibits a dynamic of gradual decrease over the three centuries. Three main blocks can be observed: the oldest period (1620–1701) has the largest mean value (2.4), while the following 150 years (second and third period) have smaller means (around 1.9) and the latest period has the lowest peak (1.6). The *σ* distribution is similar for all periods except for the oldest one. The combination of the two posterior distributions as seen in Fig 9 illustrates the interaction between *μ* and *σ*. The dispersion of the posterior distribution for the first period is much larger than the rest of the examined periods. In addition all results follow a distinctive pattern: the largest values of *σ* are only selected if the *μ* value is also large.

## Discussion

These results confirm that the original Lanchester’s laws (i.e. *linear* and *squared*) are a poor match to historical evidence. The outcome is similar to other studies, which highlighted the better match of the *logarithmic* model [51]. Beyond this replication of past results, the use of the ABC framework provides new insights to the discussion.

The decisive advantage of the *fatigue* model shows that this formulation is better supported by historical evidence than the rest of the models. The extreme psychological and physical stress conditions in the battlefield caused a gradual decrease on the efficiency of the armies. The better fit of the fourth model would suggest that this process had an impact in the final outcome. The performance of the *logarithmic* model is similar to the *fatigue* model, even though it shows slightly lower match to evidence. The explanatory power of the two classical models is much lower, as they are consistently below the best runs for any tolerance level.

The credibility of the models is not constant over the entire time span. The best matches are the oldest and more recent periods, while the third period (1793-1860) is revealed as more unpredictable. The period was dominated by the French Revolutionary Wars and the Napoleonic Wars, where traditional European tactics were transformed at a scale not previously seen. This outcome would suggest that the generalist approach undertaken by the Lanchester’s laws is not suited to study transition periods with higher rates of change.

Posterior distributions for parameters *μ* and *σ* suggest a gradual decrease of the relevance of individual fighting value. In particular, *P* values calculated for XVIIth century battles are larger and more diverse than the rest of the dataset. This result suggests that the non-professional armies of this era produced a much wider set of results under similar conditions, as the fighting value of the soldiers was much relevant than their numbers. The gradual standardisation of tactics and training would give more relevance to the size because individual fighting value was equalised between all armies. The variability of fighting value *P* within the same period is basically constant after XVIIth century. Mean values are similar for the second and third period, while showing a significant decrease after 1861. This would suggest that the evolution of warfare, now dominated by mass-production, would give even more relevance to sheer numbers while differences between individuals would then become a minor factor.

Beyond the examined scenario, the case study illustrates how *Model-Based History* could benefit from a Bayesian-inspired framework. The use of a meta-model to compute Bayes Factors allows the researcher to compare hypotheses while generating credible posterior distributions. It also shows how the original framework can be easily extended to test new hypotheses, as seen in the *fatigue* model. It is worth mentioning that Bayes Factors already take into account parsimony because complex models with larger number of parameters will generate wider posterior distributions. As a result, models with more parameters will be more times below the tolerance threshold, thus promoting simpler models.

The study of different tolerance levels also provides a cautionary tale on the use of ABC. As its name indicates it approximates the posterior distributions, and the method needs additional parameters such as the tolerance level *τ*. It means that *τ* also needs to be explored, as any other parameter. Results of the case study are a good example of the need of this exploration, as Bayes Factors for *τ* = 0.05 are radically different than the other two values. Any study using ABC should acknowledge this issue and integrate this discussion in the experiment design.

Computational models are becoming a relevant quantitative tool for historical research. This new approach allows historians to evaluate the plausibility of competing hypotheses beyond what has been discussed in natural language. It is clear that History presents a unique set of issues and challenges to formal modelling, often related to the uncertainty of the datasets collected by the researchers. In this context, the integration of model selection methods such as ABC with new datasets and computer models can provide solutions to some of the current debates of the discipline.

## Supporting Information

S1 Fig Complete parameter estimation for parameter *μ*. Parameter *μ* posterior distribution for the four models and historical periods. Results obtained from the four initial experiments with *τ* = 0.0005.
(TIFF)

S2 Fig Complete parameter estimation for parameter *σ*. Parameter *σ* posterior distribution for the four models and historical periods. Results obtained from the four initial experiments with *τ* = 0.0005.
(TIFF)

S1 File Dataset and Source code. Dataset. Dataset is distributed under the terms of a Creative Commons Attribution-ShareAlike 4.0 International License. The models and the ABC rejection algorithm were implemented in Python programming language. Source code of the model is licensed under a GNU General Public License. Last versions for both data and source code can be downloaded from https://github.com/xrubio/lanchester.
(ZIP)
