# Supplementary figures and images for: Model Selection in Historical Research Using Approximate Bayesian Computation
Source: PLoS One. 2016 Jan 5;11(1):e0146491. doi: 10.1371/journal.pone.0146491 (PMC4701162; doi:10.1371/journal.pone.0146491)

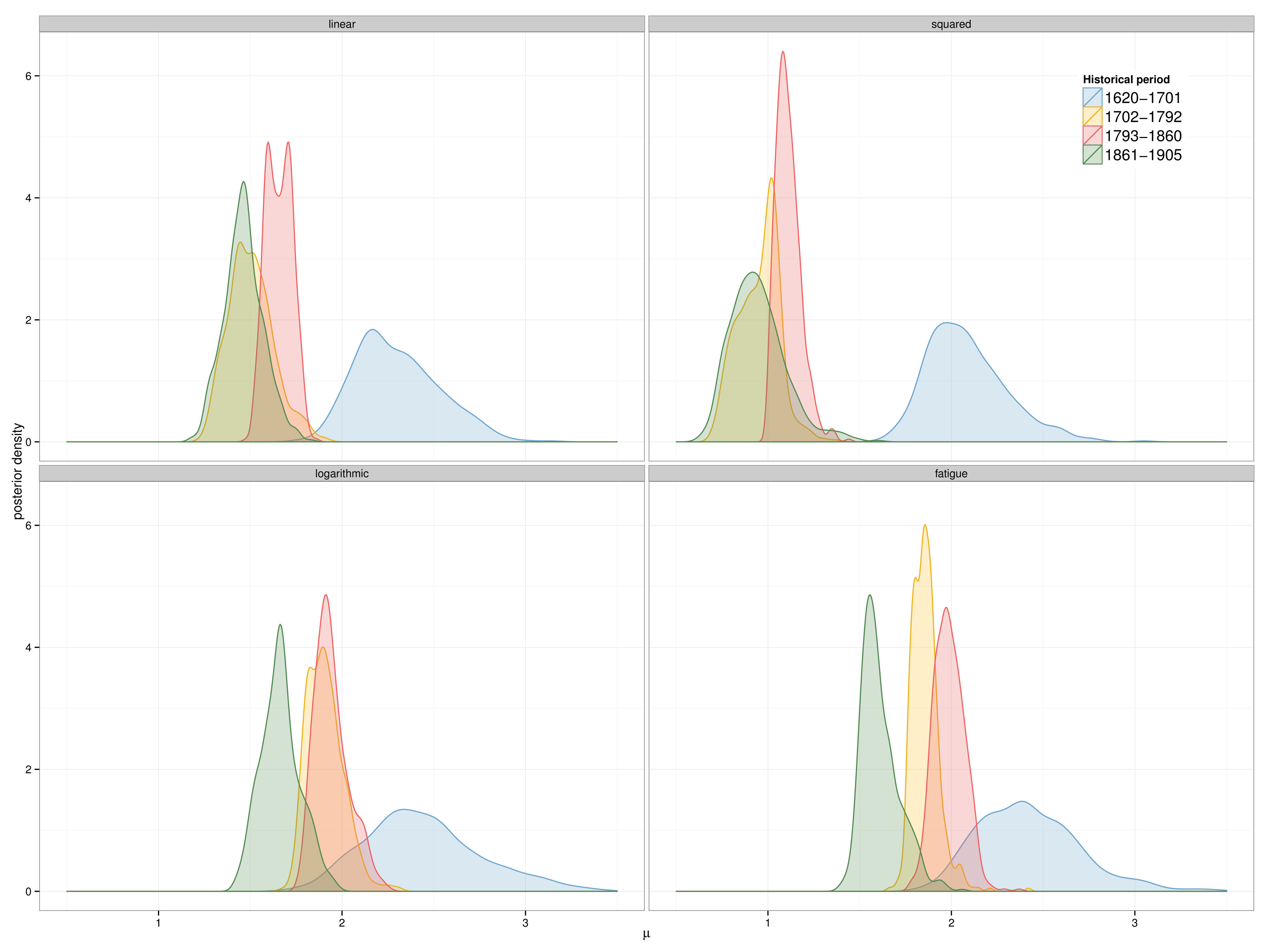

Supplement: S1 Fig — Results obtained from the four initial experiments with τ = 0.0005. (TIFF) [file pone.0146491.s001.tiff]

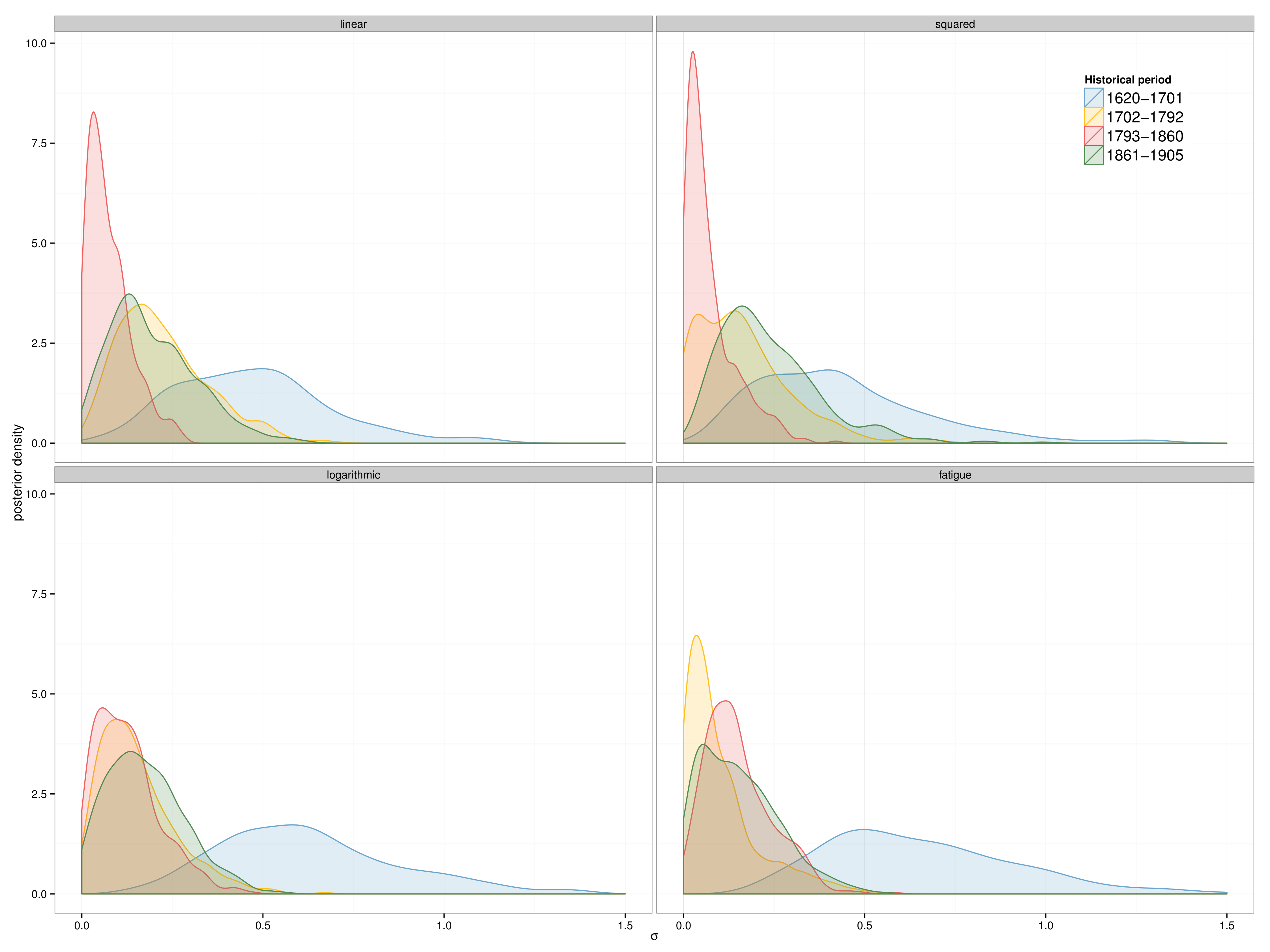

Supplement: S2 Fig — Results obtained from the four initial experiments with τ = 0.0005. (TIFF) [file pone.0146491.s002.tiff]
